# Oxygen Consumption (VO_2_) and Surface Electromyography (sEMG) during Moderate-Strength Training Exercises

**DOI:** 10.3390/ijerph19042233

**Published:** 2022-02-16

**Authors:** Muhammad Adeel, Hung-Chou Chen, Bor-Shing Lin, Chien-Hung Lai, Chun-Wei Wu, Jiunn-Horng Kang, Jian-Chiun Liou, Chih-Wei Peng

**Affiliations:** 1 International Ph.D. Program in Biomedical Engineering, College of Biomedical Engineering, Taipei Medical University, Taipei 110, Taiwan; d845108004@tmu.edu.tw; 2 School of Biomedical Engineering, College of Biomedical Engineering, Taipei Medical University, Taipei 110, Taiwan; george.jasonbiolab@gmail.com (C.-W.W.); jcliou@tmu.edu.tw (J.-C.L.); 3 Department of Physical Medicine and Rehabilitation, School of Medicine, College of Medicine, Taipei Medical University, Taipei 110, Taiwan; 10462@s.tmu.edu.tw (H.-C.C.); chlai@tmu.edu.tw (C.-H.L.); jhk@tmu.edu.tw (J.-H.K.); 4 Department of Physical Medicine and Rehabilitation, Shuang Ho Hospital, Taipei Medical University, New Taipei City 235, Taiwan; 5 Department of Computer Science and Information Engineering, National Taipei University, New Taipei City 237, Taiwan; bslin@mail.ntpu.edu.tw; 6 Department of Physical Medicine and Rehabilitation, Taipei Medical University Hospital, Taipei 110, Taiwan; 7 School of Gerontology Health Management, College of Nursing, Taipei Medical University, Taipei 110, Taiwan

**Keywords:** GEE modeling, oxygen consumption, strength training, surface electromyography

## Abstract

Oxygen consumption (VO_2_) during strength training can be predicted through surface electromyography (sEMG) of local muscles. This research aimed to determine relations between VO_2_ and sEMG of upper and lower body muscles to predict VO_2_ from sEMG during moderate-intensity strength training exercises. Of the 12 participants recruited, 11 were divided into two groups: untrained (*n* = 5; with no training experience) and trained (*n* = 6; with 2 months of training experience). On different days, each individual completed six training sessions. Each participant performed training sessions consisting of three types of dumbbell exercises: shoulder press, deadlift, and squat, while wearing a mask for indirect calorimetric measurements of VO_2_ using the Cortex Metalyzer 3B. sEMG measurements of the bilateral middle deltoid, lumbar erector spinae, quadriceps (rectus femoris), and hamstring (biceps femoris) muscles were recorded. The VO_2_ was predicted from sEMG root mean square (RMS) values of the investigated muscles during the exercise period using generalized estimating equation (GEE) modeling. The predicted models for the three types of exercises for the untrained vs. trained groups were shoulder press [QIC = 102, * *p* = 0.000 vs. QIC = 82, * *p* = 0.000], deadlift [QIC = 172, * *p* = 0.000 vs. QIC = 320, * *p* = 0.026], and squat [QIC = 76, * *p* = 0.000 vs. QIC = 348, * *p* = 0.001], respectively. It was observed that untrained vs. trained groups predicted GEE models [quasi-likelihood under an independence model criterion (QIC) = 368, *p* = 0.330 vs. QIC = 837, *p* = 0.058], respectively. The study obtained significant VO_2_ prediction models during shoulder press, deadlift, and squat exercises using the right and left middle deltoid, right and left lumbar erector spinae, left rectus femoris, and right and left biceps femoris sEMG RMS for the untrained and trained groups during moderate-intensity strength training exercises.

## 1. Introduction

According to the American College of Sports Medicine (ACSM) [1] and the American Heart Association (AHA), strength training is beneficial to one’s health [2]. It has several advantages, including increased strength and beneficial changes in body composition [1]. According to the ACSM, gaining health and fitness benefits from resistance training requires at least one set of eight to twelve repetitions of each of eight to ten exercises involving the major muscle groups [3] on two or more days per week [4].

Previous research has explored the acute metabolic demands during strength exercise. Variables such as the muscle mass [5], exercise speed [6,7,8], number of sets [9,10], number of repetitions [11,12], workload [13,14], training volume [15], and rest intervals [11,16,17] resulted in substantially greater increases in oxygen consumption (VO_2_) and energy expenditure (EE). Currently, oxygen consumption is commonly measured through indirect calorimetry, which has a stated accuracy of −2% to 4% [16]. However, acute physiological responses to skeletal muscle activation during moderate-strength exercises have not been thoroughly investigated.

Surface electromyography (EMG; sEMG), one of the most common methods of measuring muscle activation, is an electrophysiological recording technique for detecting the electric potential across muscle fiber membranes [17]. One recent study reported a relation between VO_2_ and muscle activity for squats and heel raises with 80% of one repetition maximum (1RM) in healthy male participants and observed an increase in oxygen uptake after 6 weeks of resistance exercises [18]. Another study investigated the mean correlation between the surface EMG amplitude and oxygen uptake for lower extremity muscles as 0.69~0.87 during treadmill walking in young males [19].

To the best of our knowledge, no single study has found that oxygen consumption (VO_2_) is related to the sEMG of the various upper limb and lower limb muscles during strength exercises at 60% of 1RM. It is an unexplored field of research to find which muscle sEMG has a significant association in computing oxygen consumption in healthy populations. By determining this kind of relationship, we can better explain which muscle activation can more or less predict VO_2_. Some muscles are highly active during the shoulder press, deadlift, and squat, but we also observed other muscles which are not highly active during these strength workouts.

The purpose of this study was to model oxygen consumption with the sEMG of the bilateral middle deltoid, lumbar erector spinae, quadriceps, and hamstring muscles during three dumbbell exercises (shoulder press, deadlift, and squat). The main objective of this research was to calculate the multilinear relationship between VO_2_ as a dependent variable with sEMG measurements of the bilateral middle deltoid, lumbar erector spinae, quadriceps (rectus femoris), and hamstring (biceps femoris) muscles as independent outcome measures using generalized estimating equations (GEEs).

## 2. Materials and Methods

### 2.1. Subject Recruitment

A clinical controlled trial was undertaken at Taipei Medical University Hospital, and the protocol was accepted by the TMU-Joint Institutional Review Board (IRB no.: N202004023). ClinicalTrials.gov was used to register the study (NCT04532905). Between December 2020 and May 2021, convenience sampling was used to enroll 12 young male and female volunteers into two groups. One of them was left out, and the untrained group (with no strength training experience) contained five participants and the trained group (with 2 months of strength training experience) six participants. Prior to the start of the trial, each participant signed a written consent. The research’s goals and associated risks were explained to the participants.

**Figure 1:**
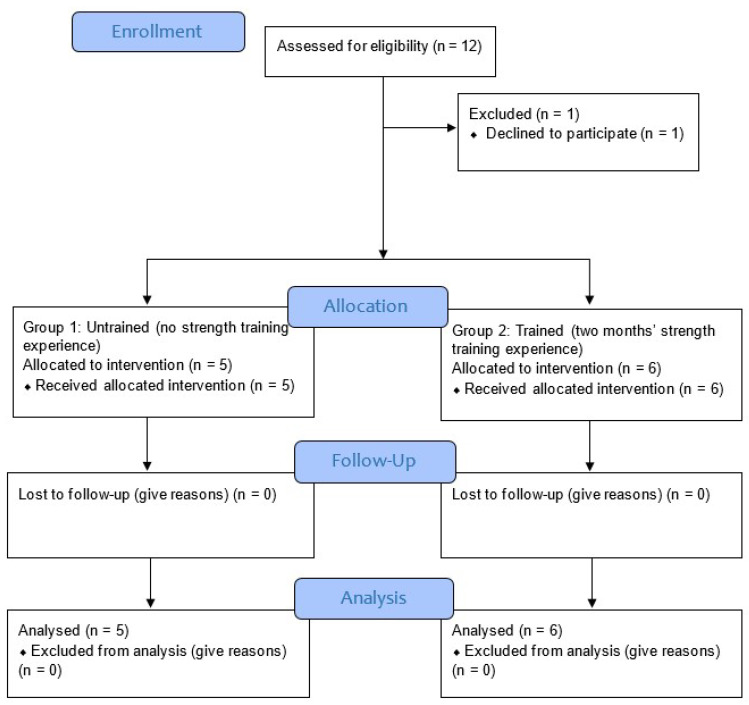
Study flow diagram [21].

The following criteria were used to include participants for the study: (1) a healthy male or female between the ages of 20 and 40; (2) without recent metabolic, systematic, or musculoskeletal disease or injury from the previous six months; (3) no recent surgical procedure that might impair workouts; (4) no medication use, particularly for sleep, depression, blood pressure control, etc.; and (5) physically fit according to the Physical Activity Readiness Questionnaire (PAR-Q) [20] (Figure 1). Experts in the field of research who were not engaged in the intervention performed the randomization, functional outcome measures, and data analysis. In this study, the order of the exercises was concealed from the participants. (Table 1).

### 2.2. Experimental Procedure

Every individual went to the exercise facility for eight separate sessions, during which they were tested, and data was collected [21]. All individuals were informed to have a meal 2~4 h before the test, to avoid alcohol and caffeine for 24 h before the test, and to avoid vigorous activity for 24~48 h before the test [22].

#### 2.2.1. Session 1

Using a Karada scan-371 body scale, the initial body weight (kg), height (cm), and body-mass index (BMI; kg/m^2^) were assessed on the first appointment (Omron, Kyoto, Japan). The PAR-Q assessed each participant’s physical fitness, and an expert researcher described the testing and training techniques to them. In this session, participants used the Cortex Metalyzer 3B (Cortex, Leipzig, Germany) to undertake an incremental cycling test while wearing a mask to measure their cardiorespiratory fitness and familiarize themselves with using a mask for workouts. Before each test, the flow and gas sensors were calibrated. The room’s temperature and humidity were set to 22~27 °C and 52~64%, respectively. During the VO_2_max testing, the individual pedaled the bicycle at 60 revolutions per minute (rpm) against 25 W of resistance. At the start of each 2 min stage, the resistance was raised by 25 W [23]. The test was terminated when two of three requirements were met: a respiratory exchange ratio (RER) of ≥ 1.1 occurred, a heart rate (HR) within 10 beats or over their theoretical aged-predicted maximal HR (220—age) was reached, or an expression of Borg rate of perceived exertion (RPE) of ≥16/20 was achieved [24].

#### 2.2.2. Session 2

Using an audible metronome, each participant did three to five sets of shoulder press, deadlift, and squat exercises using dumbbells at a tempo of 1.5 s up and 1.5 s down to attain their maximal 1RM. To account for any changes in the lifting cadence of participants, an audible metronome was utilized. Participants warmed up by performing eight to ten times with a light weight, three to five times with a moderate weight, and one to three times with a heavy weight. Following the initial sets, 1RM load was assessed by gradually rising the weight on consecutive tries until an individual was failing to carry out an attempt using appropriate technique and through the full range of motion [25]. A 2~4 min rest time was granted between each set for each participant. Between the 1RM shoulder press, deadlift, and squat, participants were given a 10~15 min rest interval during which they may walk, do minor stretches, and drink small quantities of water [26]. Participants completed three familiarization sessions on consecutive days after measuring 60% RM before commencing the regular trainings.

#### 2.2.3. Sessions 3–8

Prior to the commencement of the training session, every individual stretched and warmed up for about 10 min. All individuals did six training sessions in an alternate order (Table 1), each at the same time of day, consisting of shoulder press, deadlift, and squat exercises at 60% of 1RM, three sets of 10 repetitions at a tempo of 1.5 s concentric and 1.5 s eccentric way using a metronome to control for possible changes in the lifting speed of individuals. There was a 2 min rest break between each set and an 8 min rest period between each type of exercise. Each training session was separated by 24~48 h of rest periods.

During each training session, oxygen consumption (VO_2_) was measured using a breath-by-breath analysis on a Cortex Metalyzer 3B. Individuals of the study were given details of the Borg rate of perceived exertion (RPE) scale (6–20) before they began training. For each training session, the VO_2_, RER, and HR were measured for a total of 52:30 min, which included the resting (10 min), exercise (30 s × 9 sets), rest after exercise (2–8 min), and recovery (10 min). A portable Omron sphygmomanometer (Omron Healthcare, Lake Forest, IL, USA) and an RPE scale were used to measure blood pressure (systolic (SBP) and diastolic blood pressure (DBP)) and RPE before the beginning of a workout session and shortly after each exercise. The strength of the bilateral middle deltoid, lumbar erector spinae, quadriceps (rectus femoris), and hamstring (biceps femoris) muscles was measured two times before the first training and after the six sessions of training with a microFET3 dynamometer in newtons (N) (Hoggan Scientific, Salt Lake, UT, USA).

**Figure 2:**
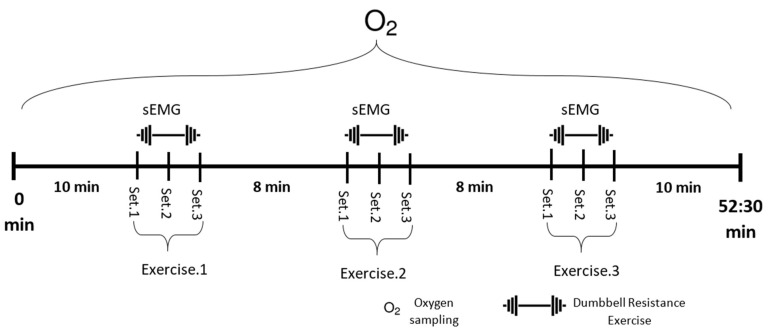
Experiment training protocol. Each exercise took 30 s with a 2 min rest interval between each set and an 8 min rest interval between each type of exercise. VO_2_ was measured during the entire session, while sEMG from eight different muscles was measured during the exercise periods.

sEMG readings were recorded for training sessions 1, 3, and 6 with Noraxon wireless sensors (Noraxon USA, Scottsdale, AZ, USA) using bipolar AgCl_2_ surface electrodes applied over eight muscles: RMD, right middle deltoid; LMD, left middle deltoid; RLES, right lumbar erector spinae; LLES, left lumbar erector spinae; RRF, right rectus femoris; LRF, left rectus femoris; RBF, right biceps femoris; and LBF, left biceps femoris. A 2 cm gap was provided between the two sensors of an electrode pair [18]. The sampling rate of the sEMG device was 1500 Hz, and raw signals during the training session were collected for each exercise set for 30 s (Figure 2).

Raw sEMG signals were treated in MATLAB R2021a (MathWorks, Natick, MA, USA) for signal analysis and processing. The raw signal for each set was filtered using a fourth-order Butterworth bandpass filter [27] (high pass with 50-Hz cutoff and low pass with 450-Hz cutoff) and smoothed, and the root mean square (RMS) was calculated. The RMS of each set of exercises was normalized by dividing the RMS value by the total training weight. The total training weight is a sum of bodyweight plus training load of every exercise [18]. The same researcher performed electrode placement throughout the training sessions.

### 2.3. Outcome Measures

The variables that were measured were: VO_2_ (ml/kg/min), sEMG (microvolts (µV)), and muscle strength (N). For each exercise of training sessions 1, 3, and 6, VO_2_ was recorded during the exercise, rest, and recovery periods, and results are presented as average values, while the normalized sEMG_rms (µV) for each exercise set was calculated.

### 2.4. Statistical Analysis

Excel software was utilized to handle raw data from the Cortex metalyzer and normalized sEMG rms (µV) from MATLAB. SPSS software (IBM SPSS Statistics vers. 19, Armonk, NY, USA) was used for statistical analysis. To confirm that the data was normal, a bell-shaped histogram was utilized. Results shows the baseline characteristics of the subjects. Study data are presented in the form of the mean ± standard deviation (SD), and the significance level was set to *p* < 0.050. The data of this research were continuous repeated measurements and generalized estimating equations (GEEs) [28]; a backward deletion approach was utilized in SPSS to model the VO_2_ of various factors [21]. VO_2_ (ml/kg/min) and normalized sEMG rms (µV) of eight muscles throughout the workout session were used in the GEE analysis. GEE models were calculated in two categories:

(1) **Group models:** Data from the exercise session, including VO_2_ (ml/kg/min) and normalized sEMG rms (µV), were used to construct group models. The exercise type (shoulder press, deadlift, and squat) was analyzed as a factor, as well as the dependent variable (VO_2_ in ml/kg/min) and covariates or independent variables (normalized sEMG_rms in µV) of eight muscles for the untrained and trained groups across each set of data.
(2) **Exercise models:** Data from the exercise phase, including VO_2_, was used to estimate exercise models. Using the same variables as described before, three types of exercise models were established. For the untrained and trained groups, the factors considered were training session (sessions 1, 3, and 6), dependent variable (VO_2_ in ml/kg/min), and covariates (normalized sEMG rms in µV) of eight muscles across each set of data.

For the GEE models, the estimate (ß), standard error (SE), 95% confidence interval (CI), and *p*-value were derived [29]. A more accurate estimate has a narrower 95% CI, whereas a less accurate estimate has a wider 95% CI [30]. The quasi-likelihood under an independent model criterion (QIC) was availed to calculate the fit of the GEE models for the group and exercise models, with a smaller QIC indicating a better model fit [31].

The difference within and between subjects for VO_2_ and normalized sEMG rms (µV) of eight muscles was presented by using a two-way repeated measure ANOVA. Pre- vs. post-exercise muscle strengths were also compared using a two-way repeated measure ANOVA. 

## 3. Results

### 3.1. Baseline Characteristics of Participants

A total of 12 individuals were recruited for this study, one of which was eliminated. 11 of the 12 individuals were divided into two groups based on their strength training experience: untrained (*n* = 5) and trained (*n* = 6). In Table 2, all participants’ baseline characteristics are reported, including age (years), gender (male/female), height (cm), body weight (kg), BMI (kg/m^2^), and 60% 1RM training weight of shoulder press, deadlift, and squat. The two groups differed in age, male/female ratio, height, body weight, BMI, and 60% 1RM training loads for the three exercises. Neither group had any side effects during or after the six workout sessions.

### 3.2. VO_2_ Models of Three Training Sessions (Group Models)

VO_2_ was predicted by the GEE model over the course of three training sessions but did not reach the level of significance. For the untrained group, right biceps femoris (RBFsEMG_rms) [*p* = 0.330; 95% CI = −0.532~1.586] predicted VO_2_, and for the trained group, left middle deltoid (LMDsEMG_rms) [*p* = 0.058; 95% CI = −0.010~0.607] predicted VO_2_ without attaining the level of significance. QIC values for the group models were 368 and 867 for the untrained and trained groups, respectively (Table 3).

### 3.3. VO_2_ Models of Three Training Sessions (Exercise Models)

For the untrained group during the shoulder press, the left biceps femoris (LBFsEMG_rms) [* *p* = 0.000; 95% CI = −27.967~−19.721], right middle deltoid (RMDsEMG_rms) [* *p* = 0.000; 95% CI = 0.543~1.341], left middle deltoid (LMDsEMG_rms) [* *p* = 0.000; 95% CI = −1.016~−0.457], right biceps femoris (RBFsEMG_rms) [* *p* = 0.000; 95% CI = 9.890~24.286], and left rectus femoris (LRFsEMG_rms) [* *p* = 0.001; 95% CI = −1.646~−0.454] significantly predicted VO_2_. For the trained group, only the left rectus femoris (LRFsEMG_rms) [* *p* = 0.000; 95% CI = 4.131~11.240] attained a significant level in predicting VO_2_. QIC values for the shoulder press models were 102 and 82 for the untrained and trained groups, respectively (Table 4a).

For the untrained group during the deadlift, the right lumbar erector spinae (RLESsEMG_rms) [* *p* = 0.000; 95% CI = 6.573~12.159], left lumbar erector spinae (LLESsEMG_rms) [* *p* = 0.000; 95% CI = −14.407~−6.448], and right biceps femoris (RBFsEMG_rms) [* *p* = 0.000; 95% CI = −2.985~−1.186] significantly predicted VO_2_. For the trained group, the left lumbar erector spinae (LLESsEMG_rms) [* *p* = 0.026; 95% CI = 0.411~6.313] attained a significant level in predicting VO_2_. QIC values for deadlift models were 172 and 320 for the untrained and trained groups, respectively (Table 4b).

For the untrained group during squat, the left biceps femoris (LBFsEMG_rms) [* *p* = 0.000; 95% CI = −12.318~−10.207], right lumbar erector spinae (RLESsEMG_rms) [* *p* = 0.000; 95% CI = −4.325~−2.312], left lumbar erector spinae (LLESsEMG_rms) [* *p* = 0.000; 95% CI = 4.108~9.675], left middle deltoid (LMDsEMG_rms) [* *p* = 0.000; 95% CI = 0.406~0.901], and right biceps femoris (RBFsEMG_rms) [* *p* = 0.000; 95% CI = 0.992~2.524] significantly computed VO_2_. For the trained group, the left lumbar erector spinae (LLESsEMG_rms) [* *p* = 0.001; 95% CI = 0.191~0.797] attained a significant level in predicting VO_2_. Values of the QIC for the squat models were 76 and 348 for the untrained and trained groups, respectively (Table 4c).

### 3.4. Comparison between VO_2_ and Normalized sEMGrms

Table 5 presents the within and between-subject comparison of different variables using repeated ANOVA. For the within-subject comparison, *p*-values were significantly different for only right lumbar erector spinae during shoulder press (SP) *p* = 0.016 * and squat (SQ) *p* = 0.023 * exercises. Meanwhile, for the between-subject comparison, oxygen consumption during shoulder press *p* = 0.005 *, right and lumbar erector spinae during deadlift (DL) *p* = 0.048 * and *p* = 0.033 *, right and left rectus femoris during squat *p* = 0.014 * and *p* = 0.032 *, and left biceps femoris during deadlift *p* = 0.045 * attained the significant level. 

The Mauchly’s sphericity reported that during shoulder press, the left lumbar erector spinae (0.002 ^j^), right biceps femoris (*p* = 0.004 ^p^) between repetitions and right middle deltoid (*p* = 0.009 ^a^), left middle deltoid (0.008 ^e^), right lumbar erector spinae (*p* = 0.012 ^f^), and left biceps femoris (*p* = 0.002 ^r^) between trainings did not show equal variance in means. The sphericity during deadlift showed no equal variance in means for the right middle deltoid (0.016 ^b^), right lumbar erector spinae (*p* = 0.037 ^g^), and left lumbar erector spinae (0.001 ^k^) between repetitions and right middle deltoid (*p* = 0.012 ^c^), right lumbar erector spinae (*p* = 0.000 ^h^), right rectus femoris (*p* = 0.019 ^n^), and left rectus femoris (*p* = 0.046 ^o^) between trainings. During squat exercise, the sphericity analysis did not report equal variance in means for the left lumbar erector spinae (0.000 ^l^) between repetitions and right middle deltoid (*p* = 0.043 ^d^), right lumbar erector spinae (*p* = 0.000 ^i^), left lumbar erector spinae (*p* = 0.000 ^m^), and right biceps femoris (*p* = 0.003 ^q^) between trainings.

### 3.5. Muscle Strength Pre vs. Post

Values of the muscle strength of the bilateral middle deltoid, lumbar erector spinae, quadriceps (rectus femoris), and hamstring (biceps femoris) are presented in Table 6. The within-subject comparison showed a significant difference for right and left lumbar erector spinae. Meanwhile, all the muscles attained the significance level (*p* < 0.050) for group comparison, except for the left middle deltoid muscle. For the untrained group, the right and left lumbar erector spinae, right and left rectus femoris, and right biceps femoris values increased after the six training sessions, while in the trained group, values of all of the muscles increased after the six training sessions, except for the left rectus femoris and right biceps femoris.

## 4. Discussion

The present study utilized GEE modeling to predict VO_2_ for three strength training exercises, including the shoulder press, deadlift, and squat with dumbbells in young participants. For the group models, the right biceps femoris predicted model for VO_2_ in the untrained group, while the left middle deltoid in the trained group without attaining the level of significance (Table 3). For the exercise models, the right and left middle deltoid, right and left biceps femoris, and left rectus femoris for the shoulder press, right and left lumbar erector spinae, and right biceps femoris for the deadlift and the right and left lumbar erector spinae, biceps femoris, and left middle deltoid for the squat significantly predicted the GEE models (Table 4a–c).

No single previous study predicted VO_2_ by sEMG of individual muscles during moderate-intensity strength exercises. Because there is not a lot of studies on GEE modeling during strength training exercises, it is difficult to compare our computed models with previously available research. One study reported the association between VO_2_ and sEMG RMS during cycling exercise and reported global sEMG measured from vastus lateralis muscle as a good predictor of energy expenditure in trained cyclists [32]. In some previous studies, one study reported the relation between VO_2_ and sEMG responses of the anterior tibialis (TA), gastrocnemius medial (MG), gastrocnemius lateral (LG), and soleus muscles during different speeds of treadmill walking in young, healthy males; correlations between VO_2_ and sEMG were 0.69~0.87 for those muscles [19]. Another study conducted on young males reported the effect of 6 weeks of strength training exercises and whole-body vibration on changes in normalized VO_2_ and sEMG; they monitored the rectus femoris muscle during squats and lateral gastrocnemius during heel raises [18].

The main goal of the present research was to calculate GEE models for VO_2_ in two categories (1) group and (2) exercise types. For the group models, none of the groups significantly predicted VO_2_ from sEMG RMS of individual muscles, but for exercise type models, the shoulder press exercise showed significant relations of the right and left middle deltoid, right and left biceps femoris, and left rectus femoris with VO_2_ for the untrained group [QIC = 102, * *p* = 0.000], while in the trained group, only the left rectus femoris [QIC = 82, * *p* = 0.000] were significantly correlated with the VO_2_. Lower QIC and significant *p*-values for the trained group [QIC = 82 vs. 102 and * *p* = 0.000 vs. * 0.000] are suggestive of a better GEE model than that for the untrained group (Table 4a). For the deadlift and squat exercises, the untrained group models were more predictive than those of the trained group [QIC = 172 vs. 320 and * *p* = 0.000 vs. * 0.026] and [QIC = 76 vs. 348 and * *p* = 0.000 vs. * 0.001] (Table 4b,c) [29,31]. The reason why the correlations between the two groups differed lies in the fact that the untrained group participants had no previous experience of strength training, and their 60% 1RM was lower, so their muscle activation occurred differently than that in the trained group. Another factor that may have affected the results was the gender because the untrained group consisted mostly of female participants and trained group males.

Pre vs. post static muscle strength is shown in Table 6. Some of the muscles’ strength increased after six training sessions, but out of the eight muscles, not a single one reached a significant level, because 2-week trainings are not enough to increase muscle strength as adaptation in strength would require about 12 or more weeks of consecutive trainings. However, this was not the concerned objective of this study. The changes in the VO_2_ and sEMG_rms after six trainings reported higher VO_2_ values in both groups, particularly during the squat and deadlift exercises, which may have been due to the greater training loads of these exercises. Meanwhile, the sEMG_rms amplitude was higher for the bilateral middle deltoid during the shoulder press, because it is the major muscle group that is active during that exercise. In the deadlift, the right and left middle deltoid, lumbar erector spinae, and biceps femoris were more active than the rectus femoris, and only the bilateral deltoid and rectus femoris were active during squatting.

Limitations: (1) Because this research’s sample size was limited (*n* = 11), generalization of the findings can only be addressed when the study procedure has been tested on a wider population. More studies with a higher sample size are required in the future to investigate these associations, which should include various body muscle strength training exercises with varying cadence and RM loads, as well as training sessions to failure. (2) In order to conduct a well-controlled research study, an adequate dietary evaluation, including BMI and dietary chart, might be incorporated to record a more sensitive link between VO_2_ and sEMG. (3) The trained group in this study had just 2 months of strength training experience; however, in the future, individuals with greater training experience might be recruited to establish the study protocol and translate the study outcomes into the general population. (4) The study reported results with mostly females in the untrained group and males in the trained group, which could have biased the results. Therefore, future studies will be conducted by controlling the gender factor to avoid such biases or may test such exercise protocols on a single gender. (5) This is one of the new ideas to use GEE modeling to relate and predict VO_2_ using localized muscular activity, so in the future, more studies of this kind may be required to confirm this study’s findings and improve exercise prescriptions for health and fitness purposes for the human population.

## 5. Conclusions

It is concluded that VO_2_ can be predicted from sEMG RMS during moderate-intensity strength training exercises. Because this study obtained significant VO_2_ prediction models during shoulder press, deadlift, and squat exercises using the right and left middle deltoid, right and left lumbar erector spinae, left rectus femoris, and right and left biceps femoris sEMG RMS for the untrained and trained groups.

Practical Implications: These kinds of correlations can help provide a deeper understanding of muscular activity and fatigue during strength training and facilitate relating and predicting metabolic parameters like VO_2_ with localized muscular activity. The exercise intensity and volume are two key parameters when designing training programs for all ages, but how various exercise programs alter relationships between oxygen consumption and muscular activity is still an area that future research needs to explore. This study tried to understand the relation between VO_2_ and sEMG during moderate-intensity strength exercise, which consisted of three different exercises in a single session. It offers another aspect of exercise prescription in rehabilitation and sports sciences to enhance one’s health and fitness of normal, athletic, and chronic disease people.

## Figures and Tables

**Table 1:** Exercise order (interval strength training).

Exercise Order	Training Session	Exercises		
		1	2	3
Sequence 1	Training 1	Shoulder press	Deadlift	Squat
	Training 2	Shoulder press	Deadlift	Squat
Sequence 2	Training 3	Deadlift	Shoulder press	Squat
	Training 4	Deadlift	Shoulder press	Squat
Sequence 3	Training 5	Squat	Shoulder press	Deadlift
	Training 6	Squat	Shoulder press	Deadlift
Each training was conducted on a different day for a total of six workouts.				

**Table 2:** Individual physical characteristics and training loads (*n* = 11).

Participant	Age (Years)	Gender	Height (cm)	Body Weight (kg)	Body-Mass Index (kg/m^2^)	Shoulder Press (60% RM)	Deadlift (60% RM)	Squat (60% RM)
**Untrained (*n* = 5)**								
S1	23	F	151	50	22	11.5	24	19
S2	21	F	158	59	23.7	9	16.5	14
S3	20	F	159	53	21	7	16.5	14
S4	21	F	165	53	19.5	9	16.5	14
S5	25	F	160	51	20.1	9	24	19
Mean ± SD	22.00 ± 1.79	--	158.60 ± 4.50	53.20 ± 3.12	21.26 ± 1.48	9.10 ± 1.43	19.50 ± 3.67	16.00 ± 2.45
**Trained (*n* = 6)**								
S1	26	M	175	92	30	16.5	29	26.5
S2	23	M	186	100	28.8	19	34	34
S3	29	F	160	55	21.6	9	34	29
S4	20	M	184	90	26.7	16.5	34	34
S5	29	F	165	58	21.5	16.5	39	34
S6	28	M	170	94	32.5	19	36.5	36.5
Mean ± SD	25.83 ± 3.34	--	173.33 ± 9.45	81.50 ± 17.96	26.85 ± 4.12	16.08 ± 3.36	34.42 ± 3.03	32.33 ± 3.44

Untrained; without strength training experience, Trained; two month’s strength training experience, SD, standard deviation; RM, repetition maximum of training weights in kilogram (kg) for both right and left sides; S, denotes participant number (untrained group *n* = 5 and trained group *n* = 6); F, female; M, male.

**Table 3:** Generalized estimating equations for oxygen consumption (VO_2_; ml/min/kg) predictions for three training sessions (*n* = 11).

Group	Model	Parameter	Estimate (ß)	SE	95% CI (Lower~Upper)	*p*
UTr	Model 1	Intercept	9.068	0.530	8.030~10.107	0.000
	(QIC 368)	RBF_sEMG-rms_	0.527	0.541	−0.532~1.586	0.330
Tr	Model 2	Intercept	11.134	0.703	9.757~12.511	0.000
	(QIC 837)	LMD_sEMG-rms_	0.298	0.157	−0.010~0.607	0.058

UTr, untrained (*n* = 5); Tr, trained (*n* = 6). SE, standard error; 95% CI, confidence interval; QIC, quasi-likelihood under an independence model criterion; RBFsEMG-rms, right biceps femoris muscle root mean square surface electromyography (sEMG); LMDsEMG-rms, left middle deltoid.

**Table 4:** Generalized estimating equations for oxygen consumption (VO_2_; mL/min/kg) estimation for three training sessions (*n* = 11). (a) Shoulder Press. (**b**) Deadlift. (**c**) Squat.

**(a)**							
**Exercise**	**Group**	**Model**	**Parameter**	**Estimate (ß)**	**SE**	**95% CI (Lower~Upper)**	***p***
Shoulder press	UTr	Model 1 (QIC 102)	Intercept	3.489	1.009	1.512~5.466	0.001
			LBF_sEMG-rms_	−23.844	2.104	−27.967~−19.721	0.000 *
			RMD_sEMG-rms_	0.942	0.204	0.543~1.341	0.000 *
			RBF_sEMG-rms_	17.088	3.673	9.890~24.286	0.000 *
			LMD_sEMG-rms_	−0.737	0.143	−1.016~−0.457	0.000 *
			LRF_sEMG-rms_	−1.050	0.304	−1.646~−0.454	0.001 *
	Tr	Model 2 (QIC 82)	Intercept	5.727	0.271	5.195~6.259	0.000
			LRF_sEMG-rms_	7.685	1.814	4.131~11.240	0.000 *
**(b)**							
**Exercise**	**Group**	**Model**	**Parameter**	**Estimate (ß)**	**SE**	**95% CI (Lower~Upper)**	***p***
Deadlift	UTr	Model 3 (QIC 172)	Intercept	11.701	1.065	9.613~13.789	0.000
			RLES_sEMG-rms_	9.366	1.425	6.573~12.159	0.000 *
			LLES_sEMG-rms_	−10.428	2.030	−14.407~−6.448	0.000 *
			RBF_sEMG-rms_	−2.086	0.459	−2.985~−1.186	0.000 *
	Tr	Model 4 (QIC 320)	Intercept	9.314	1.339	6.689~11.939	0.000
			LLES_sEMG-rms_	3.362	1.506	0.411~6.313	0.026 *
**(c)**							
**Exercise**	**Group**	**Model**	**Parameter**	**Estimate (ß)**	**SE**	**95% CI (Lower~Upper)**	***p***
Squat	UTr	Model 5 (QIC 76)	Intercept	10.328	0.875	8.612~12.043	0.000
			LBF_sEMG-rms_	−11.262	0.538	−12.318~−10.207	0.000 *
			RLES_sEMG-rms_	−3.318	0.514	−4.325~−2.312	0.000 *
			LLES_sEMG-rms_	6.891	1.420	4.108~9.675	0.000 *
			LMD_sEMG-rms_	0.653	0.126	0.406~0.901	0.000 *
			RBF_sEMG-rms_	1.758	0.391	0.992~2.524	0.000 *
	Tr	Model 6 (QIC 348)	Intercept	10.781	0.758	9.295~12.266	0.000
			LLES_sEMG-rms_	0.494	0.155	0.191~0.797	0.001 *

UTr, untrained (*n* = 5); Tr, trained (*n* = 6). * Shows a significant difference *p* < 0.050. SE, standard error; 95% CI, confidence interval; QIC, quasi-likelihood under an independence model criterion; Root mean square surface electromyography (sEMG) of RMD_sEMG-rms_, right middle deltoid; LMD_sEMG-rms_, left middle deltoid; RLES_sEMG-rms_, right lumbar erector spinae; LLES_sEMG-rms_, left lumbar erector spinae; RBF_sEMG-rms_, right biceps femoris; LBF_sEMG-rms_, left biceps femoris; RRF_sEMG-rms_, right rectus femoris; LRF_sEMG-rms_, left rectus femoris.

**Table 5:** Two-way repeated measures ANOVA three exercises (*n* = 11).

Parameters		Untrained (*n* = 5)	Trained (*n* = 6)	*p*-Value				
				Within Subject		Mauchly’s Sphericity		Between Groups
				Rep	Tra	Rep	Tra	
Oxygen Consumption	**SP**	4.61 ± 0.48	7.04 ± 0.44	0.372	0.909	0.724	0.369	0.005 *
	**DL**	9.17 ± 0.80	10.81 ± 0.73	0.687	0.120	0.870	0.104	0.165
	**SQ**	9.35 ± 0.76	11.60 ± 0.70	0.254	0.058	0.909	0.478	0.057
Right Middle Deltoid	**SP**	3.45 ± 0.68	3.23 ± 0.62	0.860	0.570	0.832	0.009 ^a^	0.817
	**DL**	1.06 ± 0.41	0.80 ± 0.37	0.555	0.214	0.016 ^b^	0.012 ^c^	0.653
	**SQ**	2.18 ± 0.56	1.65 ± 0.51	0.493	0.826	0.108	0.043 ^d^	0.505
Left Middle Deltoid	**SP**	2.61 ± 0.57	2.31 ± 0.52	0.105	0.574	0.235	0.008 ^e^	0.708
	**DL**	1.11 ± 0.42	0.75 ± 0.38	0.939	0.161	0.106	0.070	0.537
	**SQ**	2.04 ± 0.50	1.74 ± 0.46	0.546	0.284	0.285	0.240	0.661
Right Lumbar Erector Spinae	**SP**	0.08 ± 0.02	0.08 ± 0.02	0.815	0.016 *	0.958	0.012 ^f^	0.989
	**DL**	0.66 ± 0.08	0.42 ± 0.07	0.433	0.126	0.037 ^g^	0.000 ^h^	0.048 *
	**SQ**	0.59 ± 0.07	0.39 ± 0.06	0.630	0.023 *	0.435	0.000 ^i^	0.056
Left Lumbar Erector Spinae	**SP**	0.07 ± 0.02	0.08 ± 0.01	0.690	0.346	0.002 ^j^	0.063	0.814
	**DL**	0.71 ± 0.08	0.44 ± 0.07	0.027	0.137	0.001 ^k^	0.065	0.033 *
	**SQ**	0.59 ± 0.08	0.48 ± 0.07	0.420	0.519	0.000 ^l^	0.000 ^m^	0.314
Right Rectus Femoris	**SP**	0.12 ± 0.03	0.16 ± 0.03	0.272	0.365	0.696	0.441	0.450
	**DL**	0.45 ± 0.08	0.33 ± 0.08	0.190	0.138	0.076	0.019 ^n^	0.333
	**SQ**	1.58 ± 0.17	0.89 ± 0.15	0.297	0.407	0.326	0.675	0.014 *
Left Rectus Femoris	**SP**	0.15 ± 0.04	0.17 ± 0.04	0.056	0.872	0.409	0.531	0.676
	**DL**	0.39 ± 0.08	0.28 ± 0.07	0.189	0.368	0.569	0.046 ^o^	0.325
	**SQ**	1.46 ± 0.16	0.91 ± 0.14	0.595	0.249	0.553	0.209	0.032 *
Right Biceps Femoris	**SP**	0.04 ± 0.01	0.03 ± 0.01	0.672	0.504	0.004 ^p^	0.126	0.719
	**DL**	0.64 ± 0.09	0.42 ± 0.08	0.477	0.724	0.159	0.692	0.087
	**SQ**	0.47 ± 0.08	0.31 ± 0.07	0.136	0.162	0.447	0.003 ^q^	0.178
Left Biceps Femoris	**SP**	0.03 ± 0.01	0.03 ± 0.01	0.442	0.233	0.363	0.002 ^r^	0.618
	**DL**	0.69 ± 0.09	0.41 ± 0.08	0.458	0.357	0.105	0.514	0.045 *
	**SQ**	0.46 ± 0.06	0.31 ± 0.06	0.104	0.286	0.497	0.873	0.092

Rep, number of repetitions; Tra, number of trainings. Mean ± standard error; level of significance, * p < 0.05. SP, shoulder press; DL, deadlift; SQ, squat. ^a^ For right middle deltoid, Mauchly’s sphericity *p* = 0.009 for training, the assumption for the difference in equal variance was not met. Greenhouse-Geisser Epsilon was 0.59, which is lower than 0.75, and after correction, sphericity assumed for training did not reach the significance level, *p* = 0.888. ^b,c^ Mauchly’s sphericity *p* = 0.016 and 0.012 for repetitions and training, the assumption for the difference in equal variance was not met. Greenhouse-Geisser Epsilon was 0.61 and 0.60, which are lower than 0.75, and after correction, sphericity assumed for repetitions and training did not reach significance level *p* = 0.794 and *p* = 0.044, respectively. ^d^ Mauchly’s sphericity *p* = 0.043 for training, the assumption for the difference in equal variance was not met. Greenhouse-Geisser Epsilon was 0.65, which is lower than 0.75, and after correction, sphericity assumed did not reach the significant level, *p* = 0.846. ^e^ For left middle deltoid, Mauchly’s sphericity *p* = 0.008 for training, the assumption for the difference in equal variance was not met, and Greenhouse-Geisser Epsilon was 0.58, which is lower than 0.75, and after correction, sphericity assumed for training did not reach significant level, *p* = 0.511. ^f^ For right lumbar erector spinae, Mauchly’s sphericity *p* = 0.012 for training, the assumption for the difference in equal variance was not met. Greenhouse-Geisser Epsilon was 0.60, lower than 0.75, and after correction, sphericity did not reach significant level, *p* = 0.194. ^g,h^ Mauchly’s sphericity *p* = 0.037 and 0.000 for repetition and training, the assumption for the difference in equal variance was not met. Greenhouse-Geisser Epsilon were 0.64 and 0.52, which are lower than 0.75, and after correction, sphericity assumed did not reach the significant level *p* = 0.760 and *p* = 0.703, respectively. ^i^ Mauchly’s sphericity *p* = 0.000 for training, the assumption for the difference in equal variance was not met. Greenhouse-Geisser Epsilon was 0.52, lower than 0.75, and after correction, sphericity assumed did not reach the significant level, *p* = 0.269. ^j^ For left lumbar erector spinae, Mauchly’s sphericity *p* = 0.002 for repetition, assumption for difference in equal variance not met. Greenhouse-Geisser Epsilon was 0.56, which is lower than 0.75, and after correction, sphericity assumed did not reach significant level, *p* = 0.714. ^k^ Mauchly’s sphericity *p* = 0.001 for repetition, the assumption for the difference in equal variance was not met. Greenhouse-Geisser Epsilon was 0.55, which is lower than 0.75, and after correction, sphericity assumed did not reach significant level *p* = 0.524. ^l,m^ Mauchly’s sphericity *p* = 0.000 for repetition and training, assumption for the difference in equal variance not met. Greenhouse-Geisser Epsilon was 0.53 and 0.50, which were lower than 0.75, and after correction, sphericity assumed did not reach significant levels *p* = 0.155 and *p* = 0.624, respectively. ^n^ For right rectus femoris, Mauchly’s sphericity *p* = 0.019 for training, the assumption for the difference in equal variance was not met. Greenhouse-Geisser Epsilon was 0.62, which is lower than 0.75, and after correction, sphericity assumed did not reach significant level *p* = 0.112. ^o^ For left rectus femoris, Mauchly’s sphericity *p* = 0.046 for training, the assumption for difference in equal variance was not met. Greenhouse-Geisser Epsilon was 0.65, which is lower than 0.75, and after correction, sphericity assumed did not reach significant level *p* = 0.143. ^p^ For right biceps femoris, Mauchly’s sphericity *p* = 0.004 for repetition, the assumption for difference in equal variance was not met. Greenhouse-Geisser Epsilon was 0.57, which is lower than 0.75, and after correction, sphericity assumed did not reach significant level *p* = 0.556. ^q^ Mauchly’s sphericity *p* = 0.003 for training, the assumption for the difference in equal variance was not met. Greenhouse-Geisser Epsilon was 0.56, which is lower than 0.75, and after correction, sphericity assumed reached significant level *p* = 0.027. ^r^ For left biceps femoris, Mauchly’s sphericity *p* = 0.002 for training, the assumption for the difference in equal variance was not met. Greenhouse-Geisser Epsilon was 0.56, which is lower than 0.75, and after correction, sphericity assumed did not reach significant level *p* = 0.296.

**Table 6:** Two-way repeated measures ANOVA for manual muscle strength (MMT) (*n* = 11).

Parameters	Untrained (*n* = 5)	Trained (*n* = 6)	*p*-Value	
			Within Subject Pre vs. Post	Between Groups
Right Middle Deltoid	85.08 ± 22.01	156.28 ± 20.09	0.926	0.041 *
Left Middle Deltoid	85.49 ± 17.82	136.28 ± 16.27	0.951	0.065
Right Lumbar Erector Spinae	130.35 ± 23.14	212.27 ± 21.12	0.016 *	0.028 *
Left Lumbar Erector Spinae	134.93 ± 18.87	207.78 ± 17.23	0.017 *	0.019 *
Right Rectus Femoris	281.61 ± 34.25	426.36 ± 31.27	0.170	0.012 *
Left Rectus Femoris	283.40 ± 34.01	408.43 ± 31.04	0.085	0.024 *
Right Biceps Femoris	208.81 ± 19.06	249.48 ± 17.40	0.569	0.149
Left Biceps Femoris	181.45 ± 22.73	256.76 ± 20.75	0.950	0.037 *

Mean ± standard error; participants; *n* = 11. Muscle strength was assessed through a dynamometer in newtons (N) two times before and after six training sessions. * Shows a significant difference *p* < 0.050. Mauchly’s sphericity and Greenhouse-Geisser Epsilon were equal to 1 for every muscle, so the assumption for the difference in equal variance was met.

## Data Availability

The original contributions presented in this study are included in the article. Further inquiries can be directed to the corresponding author.

## Abbreviations

ACSM: American College of Sports Medicine; AHA, American Heart Association; ß, estimate; BMI, body-mass index; BP, blood pressure; cm, centimeter; CI, confidence interval; DBP, diastolic blood pressure; ∆d, difference; EE, energy expenditure; GEE, generalized estimating equation; HR, heart rate; IRB, institutional review board; kg, kilogram; LBF, left biceps femoris; LG, lateral gastrocnemius; LLES, left lumbar erector spinae; LMD, left middle deltoid; LRF, left rectus femoris; MG, medial gastrocnemius; ml/kg/min, milliliter per kilogram per minute; n, number of participants; N, newtons; PAR-Q, Physical Activity Readiness Questionnaire; QIC, quasi-likelihood under an independence model criterion; RBF, right biceps femoris; RER, respiratory exchange ratio; RLES, right lumbar erector spinae; RM, repetition maximum; RMD, right middle deltoid; RMS/rms, root mean square; RPE, Borg rate of perceived exertion; RPM, revolutions per minute; RRF, right rectus femoris; SD, standard deviation; SE, standard error; sEMG, surface electromyography; SBP, systolic blood pressure; SPSS, Statistical Package for the Social Sciences; TA, tibialis anterior; Tr, trained; UTr, untrained; µV, microvolt; VO_2_, oxygen consumption.
