# Correction: Adeel et al. Oxygen Consumption (VO_2_) and Surface Electromyography (sEMG) during Moderate-Strength Training Exercises. *Int. J. Environ. Res. Public Health* 2022, *19*, 2233

**DOI:** 10.3390/ijerph22111754

**Published:** 2025-11-20

**Authors:** Muhammad Adeel, Hung-Chou Chen, Bor-Shing Lin, Chien-Hung Lai, Chun-Wei Wu, Jiunn-Horng Kang, Jian-Chiun Liou, Chih-Wei Peng

**Affiliations:** 1 International Ph.D. Program in Biomedical Engineering, College of Biomedical Engineering, Taipei Medical University, Taipei 110, Taiwan; d845108004@tmu.edu.tw; 2 School of Biomedical Engineering, College of Biomedical Engineering, Taipei Medical University, Taipei 110, Taiwan; george.jasonbiolab@gmail.com (C.-W.W.); jcliou@tmu.edu.tw (J.-C.L.); 3 Department of Physical Medicine and Rehabilitation, School of Medicine, College of Medicine, Taipei Medical University, Taipei 110, Taiwan; 10462@s.tmu.edu.tw (H.-C.C.); chlai@tmu.edu.tw (C.-H.L.); jhk@tmu.edu.tw (J.-H.K.); 4 Department of Physical Medicine and Rehabilitation, Shuang Ho Hospital, Taipei Medical University, New Taipei City 235, Taiwan; 5 Department of Computer Science and Information Engineering, National Taipei University, New Taipei City 237, Taiwan; bslin@mail.ntpu.edu.tw; 6 Department of Physical Medicine and Rehabilitation, Taipei Medical University Hospital, Taipei 110, Taiwan; 7 School of Gerontology Health Management, College of Nursing, Taipei Medical University, Taipei 110, Taiwan

In the original publication [1], Reference 21 was not cited in Figure 1’s caption. The citation has now been inserted and should read:

**Figure 1.** Study flow diagram [21].

The Data Availability Statement was updated to “The original contributions presented in this study are included in the article. Further inquiries can be directed to the corresponding author.”

The authors state that the scientific conclusions are unaffected. This correction was approved by the Academic Editor. The original publication has also been updated.
